# Dynamic alterations in the lung microbiota in a rat model of lipopolysaccharide-induced acute lung injury

**DOI:** 10.1038/s41598-022-08831-8

**Published:** 2022-03-21

**Authors:** Zhigang Tian, Enqi Wu, Jia You, Gang Ma, Shenzhen Jiang, Yuanyuan Liu, Jia Hou, Sihan Hou, Yaqin Ling, Lingpeng Pei, Xiwei Zheng

**Affiliations:** 1 Department of Respiratory and Critical Care Medicine, General Hospital of Ningxia Medical University, No. 804 Shenglijie, Xingqing District, Yinchuan, 750004 China; 2 Key Laboratory of Ethnomedicine (Minzu University of China), Ministry of Education, Minzu University of China, No. 27 Zhongguancun South Avenue, Beijing, 100081 China; 3 Biotherapy Center, The Seventh Medical Center of PLA General Hospital, Beijing, China

**Keywords:** Microbiology, Physiology, Diseases

## Abstract

The lung microbiota have been found to be substantially altered in numerous pulmonary disorders, and crosstalk between the host pathophysiology and lung microbiota plays critical roles in the regulation of disease states. The aim of this study was to investigate dynamic changes in the lung microbiota during different stages of acute lung injury and acute respiratory distress syndrome (ALI/ARDS). Rats receiving an intraperitoneal administration of lipopolysaccharide (LPS) were sacrificed at 12 and 48 h after injection, and the hematological parameters, serum cytokine levels, and histological characteristics of the lung tissue and lung microbiota were assessed. After LPS injection, along with fluctuations of systemic cytokine levels and the onset and regression of pulmonary edema, the diversity, components, and functionalities of the pulmonary microbiota underwent significant dynamic changes. The volatility of the α-diversity indices narrowed after LPS injection, and the indices significantly decreased 48 h later. The abundance of 18 genera and functionality of adenosine triphosphate–binding cassette (ABC) transporters, pentose phosphate, and bacterial chemotaxis pathways were found to significantly differ between specified time points. Several significant correlations between the components and functionalities of the lung microbiota and indicative symptoms of ALI/ARDS were also observed. *Brevibacterium* was correlated with cytokines tumor necrosis factor (TNF)-α, interleukin (IL)-10, and IL-6 and with hematological percentage of neutrophils (NEU%); Wnt, Notch, and chronic myeloid leukemia signaling pathways were correlated with IL-1β; mitogen-activated protein kinase (MAPK) signaling pathway–yeast was correlated with IL-10; and the pathways of ascorbate and aldarate metabolism and basal transcription factors were correlated with platelet-related indicators. The correlations between the lung microbiota and indicative symptoms of ALI/ARDS identified in this study support further investigation into the underlying mechanism of host–microbiota interactions during lung injury and repair.

## Introduction

In recent years, with the widespread application of culture-independent, second-generation sequencing methods in microbiota research, the role of the microbiota in various human habitats in maintaining normal homeostasis and regulating disease states has attracted increasing attention^1^. The lungs, previously considered sterile, have been shown to have a diverse and dynamic bacterial community^2^. Although a relative paucity of data on the physiological implications of the respiratory microbiome exists in comparison to research involving the gut microbiome, the lung microbiota have been found to be substantially altered in the context of numerous respiratory disorders^3^. Studies have reported that the lung microbiota are strongly correlated with alterations of both systemic and alveolar immunity^4,5^. On one hand, the host inflammatory response can significantly change the lung microbial community by altering the lung microenvironment, which is conducive to the proliferation of certain microbial species^5^; on the other hand, such changes can result in greater susceptibility to several lung diseases, such as infections by various pathogens, lung injury, and asthma^5–7^. Growing evidence for the correlation between variations in the lung microbiota and the physiological severity of pulmonary disease and response to therapy^8–10^ indicates that the lung microbiome contains unexplored therapeutic targets for the prevention and treatment of lung disease.

Application of lipopolysaccharide (LPS) can stably trigger a systemic immune response in experimental animals and cause lung histological changes, such as severe epithelial and endothelial damage, inflammatory cell infiltration into the lung, and accumulation of protein-rich edema fluid in alveolar spaces^11–13^. Therefore, LPS-induced acute lung injury and acute respiratory distress syndrome (ALI/ARDS) has become one of the most widely used in vivo models for studying the molecular mechanisms of inflammation-associated lung injury and researching pharmacological treatments thereof. However, the dynamic impact of the changes of inflammatory cells and immune factors on the pulmonary microbiota during the occurrence and development of LPS-induced ALI/ARDS is still lacking.

In this study, we present for the first time the dynamic effects of single-dose intraperitoneal (i.p.) administration of LPS on the lung microbiota. Herein, we also analyzed the association between the lung microbiota and other quantitative ALI/ARDS symptom indicators, including blood cell count and serum cytokine levels.

## Results

### Histopathological characteristics and W/D ratio

**Figure 1:**
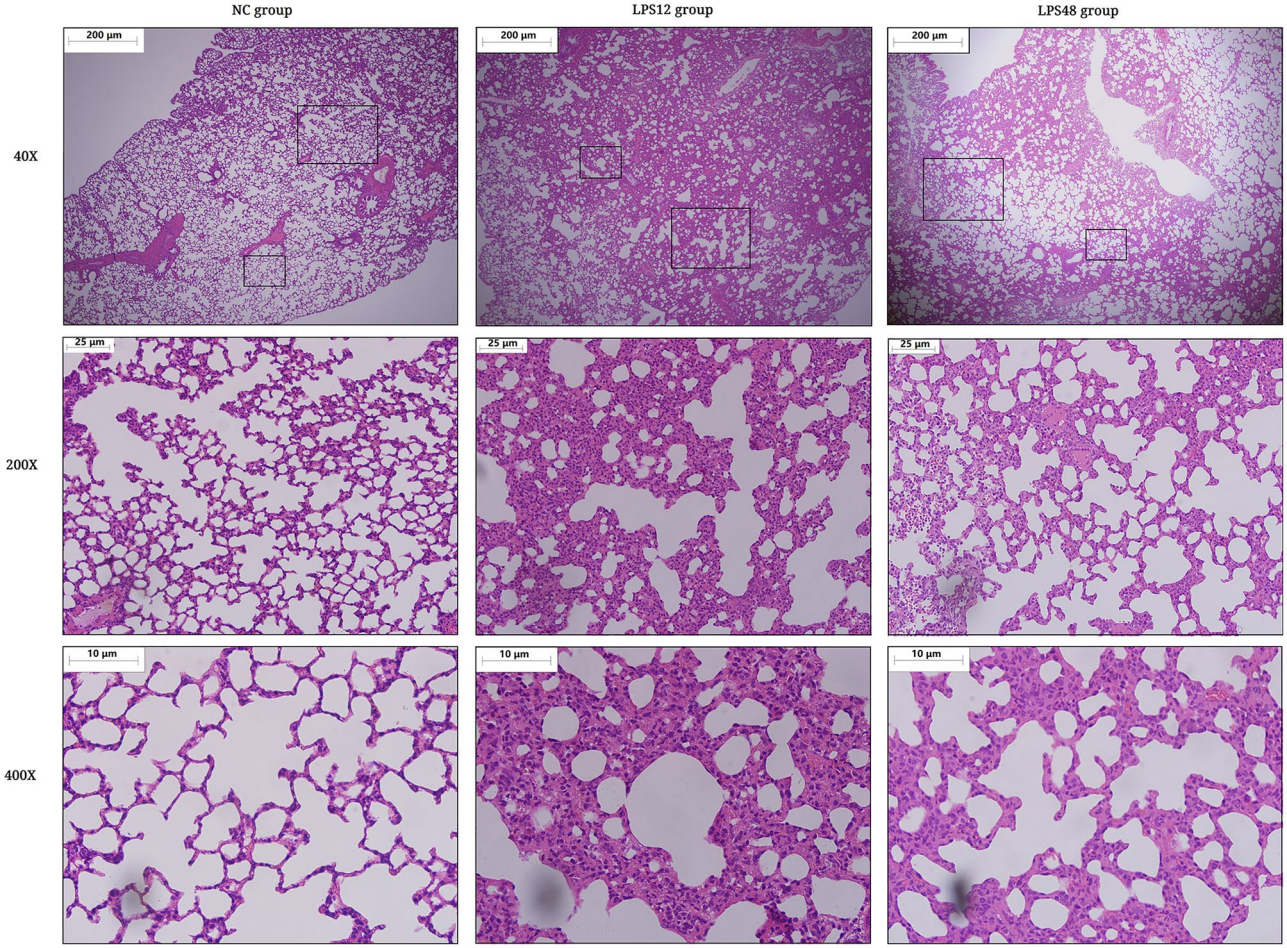
Representative H&E-stained lung sections showing morphological changes in lung tissue. The Control group presented normal architecture with no damage to the lung tissue. In the LPS12 group, the tissue showed interalveolar septum thickening, irregular distribution of air spaces, pulmonary interstitial edema, and inflammatory cell infiltration. In the LPS48 group, the tissue still showed perivascular and peribronchial inflammatory infiltration, but the alveolar architecture and histological changes were slightly relieved.

The H&E-stained sections of lung tissue of the Control group had normal architecture with no signs of inflammation. However, tissue from the LPS12 group showed interalveolar septum thickening, irregular distribution of air spaces, pulmonary interstitial edema, and inflammatory cell infiltration with a predominance of polymorphonuclear cells. In the LPS48 group, although the alveolar architecture and histological changes were slightly relieved, the lung tissue still showed perivascular and peribronchial inflammatory infiltration with inflammation in the alveolar septa, and we also observed patchy intra-alveolar and interstitial hemorrhage (Fig. 1).

**Figure 2:**
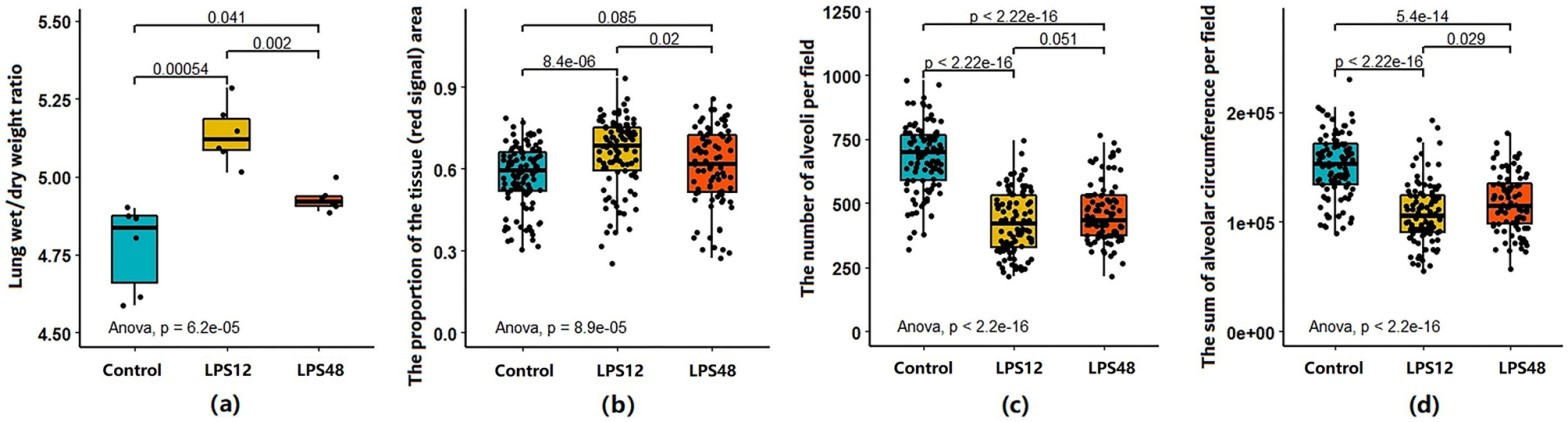
Changes in the wet/dry (W/D) ratio and quantified histological characteristics at indicated time points after LPS injection. (**a**) The W/D ratio was measured to estimate the level of pulmonary edema. (**b**) The proportion of the area of the tissue (red signal) representing the ratio of infiltration to air space was calculated for each screened image in order to quantify the level of interalveolar septum thickening. (**c**) The number of alveoli per field was calculated based on the recognition of the area of white signal in each screened image. (**d**) The sum of the alveolar circumferences was calculated based on the inner space peripheral length of each empty alveolus recognized by ImagePro Plus software version 5.0.

We estimated the volume of lung water in each group by the wet/dry (W/D) ratio. Compared with the Control group, the W/D ratio increased significantly in the LPS12 group, suggesting the occurrence of pulmonary edema. The ratio was reduced significantly in the LPS48 group as compared with the LPS12 group, but it was still significantly higher than that of the Control group (Fig. 2a).

Quantification of histological changes revealed similar results. The proportion of the tissue area (red signal) of the LPS12 group was significantly higher than that of the Control group and was decreased significantly in the LPS48 group (Fig. 2b). In addition, the alveolar count (Fig. 2c) and circumferential measurement (Fig. 2d) showed that as compared with the Control group, the number of alveoli used for effective gas exchange and the area of gas exchange were significantly reduced after LPS injection. Furthermore, as time progressed, the LPS48 group showed signs that these symptom indicators were relieved, compared with the LPS12 group.

### Routine blood examination and serum cytokine measurement

**Figure 3:**
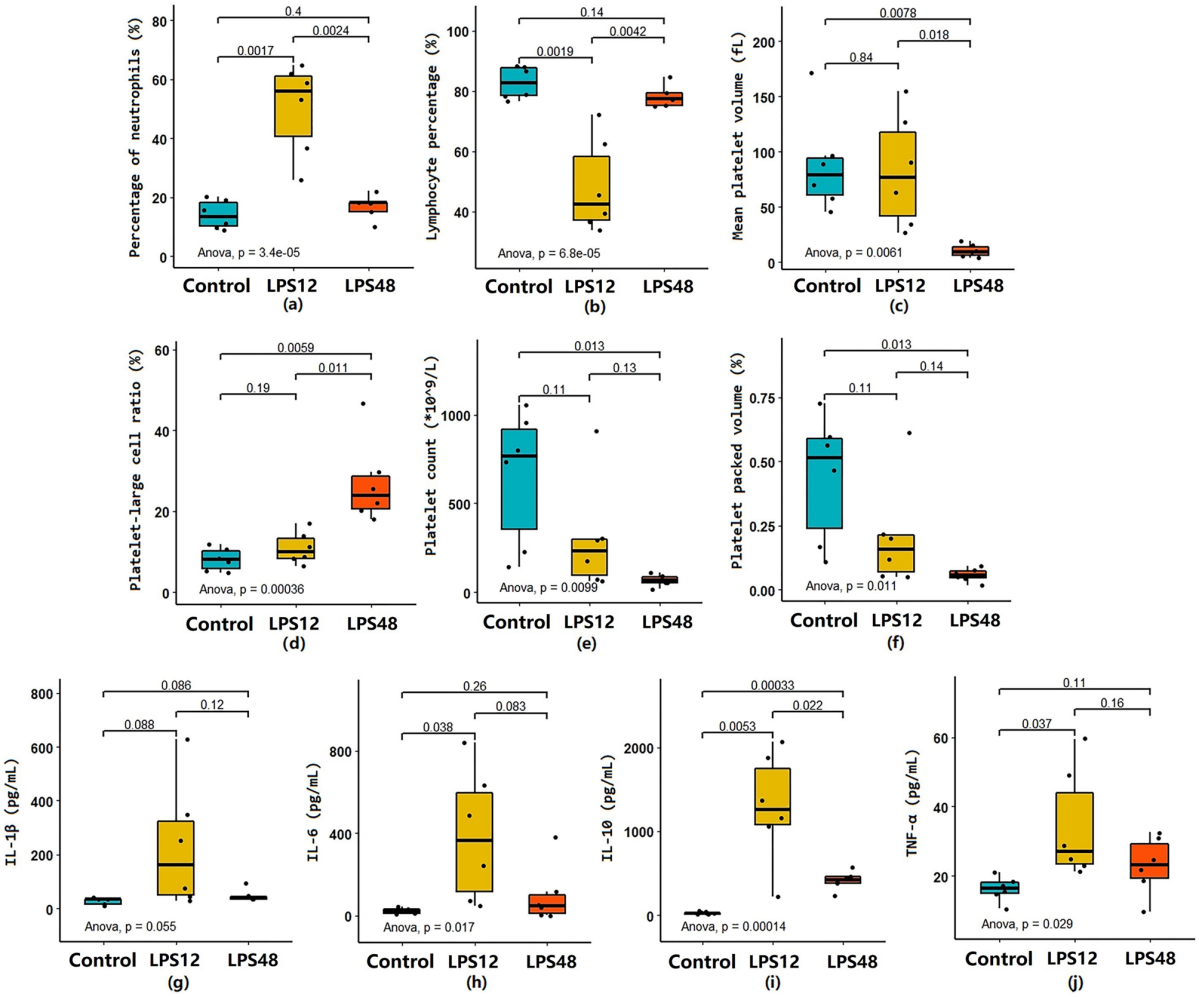
Changes in the routine blood examination parameters and serum cytokine values at indicated time points after LPS injection. Of the 24 hematological parameters examined, six—(**a**) percentage of neutrophils (NEU%), (**b**) lymphocyte percentage (LYM%), (**c**) mean platelet volume (MPV), (**d**) platelet/large-cell ratio (PLCR), (**e**) platelet count (PLT), and (**f**) platelet packed volume (PCT)—were significantly changed after LPS challenge. Serum levels of four cytokines—(**g**) IL-1β, (**h**) IL-6, (**i**) IL-10, and (**j**) TNF-α—were likewise found to have significantly changed. The 24 hematological parameters were white blood cell count, LYM%, percentage of monocytes, NEU%, percentage of eosinophils, percentage of basophils, absolute lymphocyte value, absolute value of monocytes, absolute value of neutrophils, absolute eosinophils, basophil absolute value, red blood cell (RBC) count, hemoglobin (HB), hematocrit, mean RBC volume, average RBC HB content, mean RBC HB concentration, RBC distribution width coefficient of variation (CV), RBC distribution width SD, PLT, platelet distribution width, MPV, PCT, and PLCR.

Altogether, we captured 24 hematological parameters in this study. Of these, six were found to have significantly changed after LPS challenge. As shown in Fig. 3, the percentage of neutrophils (NEU%) significantly increased 12 h after LPS injection and returned to a level close to that of the Control group 48 h after LPS injection. Although lymphocyte percentage (LYM%) showed the opposite trend, it was significantly decreased at the 12-h time point, and it returned to normal at the 48-h time point. Mean platelet volume (MPV) and platelet–large-cell ratio (PLCR) gradually increased, while platelet count (PLT) and platelet packed volume (PCT) significantly decreased, as time progressed after LPS injection.

We also analyzed levels of interleukin (IL)-1β, IL-6, IL-10, and tumor necrosis factor (TNF)-α in serum samples. The levels of all four cytokines increased at 12 h after LPS injection and were normalized at 48 h (Fig. 3g–j).

### Overall assessment of lung microbiota

**Figure 4:**
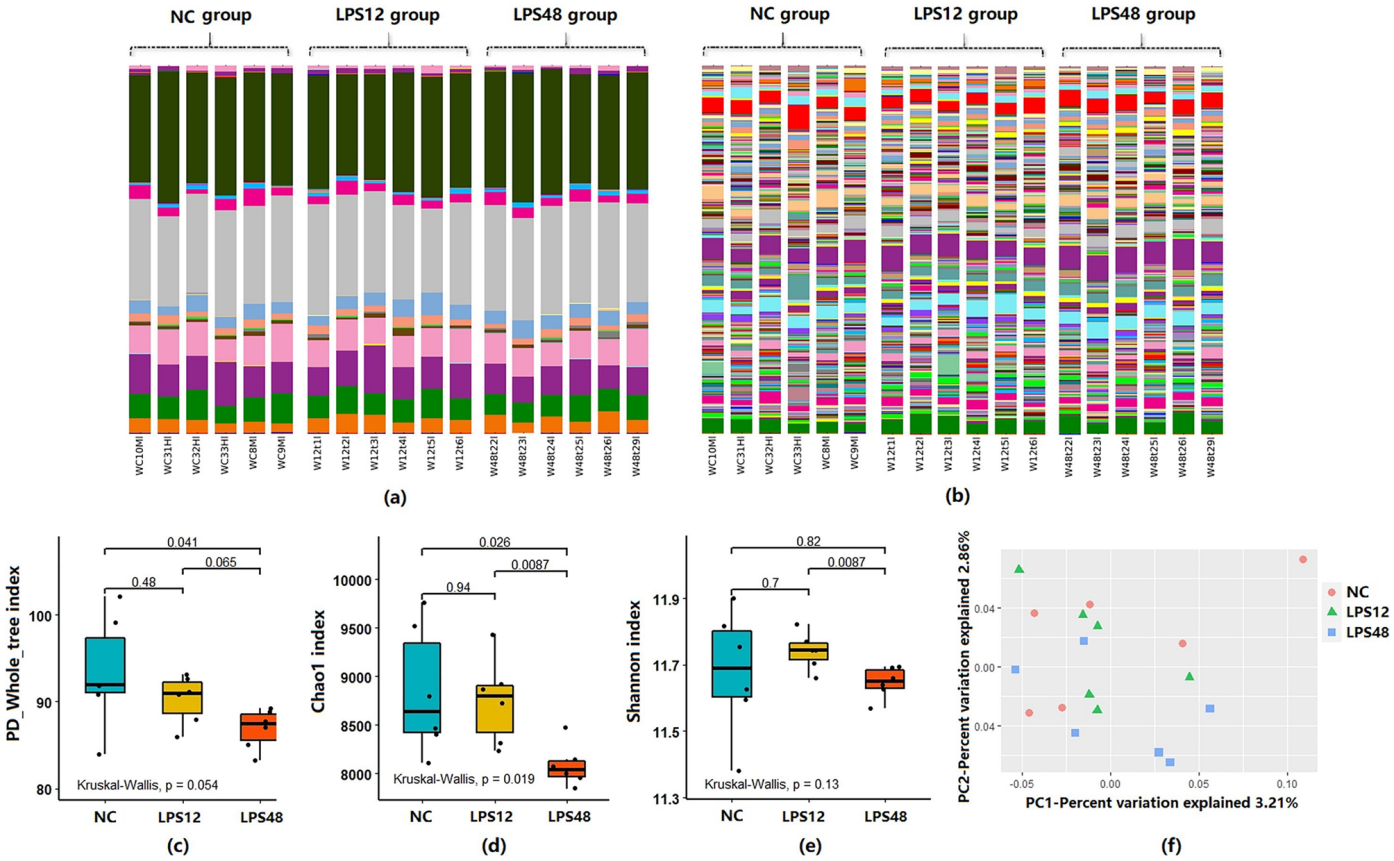
Overall assessment of the microbiota in lung tissue. Bar plots of the lung microbiota components in each sample in each of the three groups. Annotated at (**a**) phylum level; (**b**) genus level. In our comparison of α-diversity values of the lung microbial communities among the three groups, the (**c**) PD whole tree, (**d**) Chao1, and (**e**) Shannon α-diversity indices of all narrowed after LPS injection and decreased significantly in the LPS48 group. (**f**) Two-dimensional PCoA plots showing differences in the lung microbiota of rats from the three groups based on the Bray–Curtis distance metric. *P* = 0.002 and *r*^2^ = 0.1263 in the Adonis test; *P* = 0.002 and adjusted *r*^2^ = 0.1258 in db-RDA.

We ultimately generated a total of 24,317 zero-radius operational taxonomic units (ZOTUs) from 864,727 high-quality reads. A total of 23,459 ZOTUs were successfully annotated by the Ribosomal Database Project (RDP) classifier, comprising 35 phyla, 79 classes, 137 orders, 270 families, and 582 genera (Fig. 4a,b).

### Comparison of lung microbiota diversity

We compared the three α-diversity indices of PD whole tree, Chao1, and Shannon between the groups. After LPS injection, the variation ranges of all three indices narrowed (Fig. 4c–e). A non-parametric test of differences between groups found that these indices did not significantly differ between the Control and LPS12 groups; however, the LPS48 group showed different degrees of decline in all three indices as compared with the other two groups. In the LPS48 group, the PD whole tree index was significantly lower than in the Control group, the Chao1 index was significantly lower than in both the Control and LPS12 groups, and the Shannon index was significantly lower than in the LPS12 group.

We created a Bray–Curtis distance metric from the subsampled ZOTU table to explore structural differences in bacterial communities among the three groups. Principal coordinate analysis (PCoA) showed partial separation of microbiota communities by group. Adonis tests and distance-based redundancy analysis (db-RDA) both confirmed a significant association between the different treatment groups and bacterial-community structures (Fig. 4f).

### Comparison of lung microbiota components

**Figure 5:**
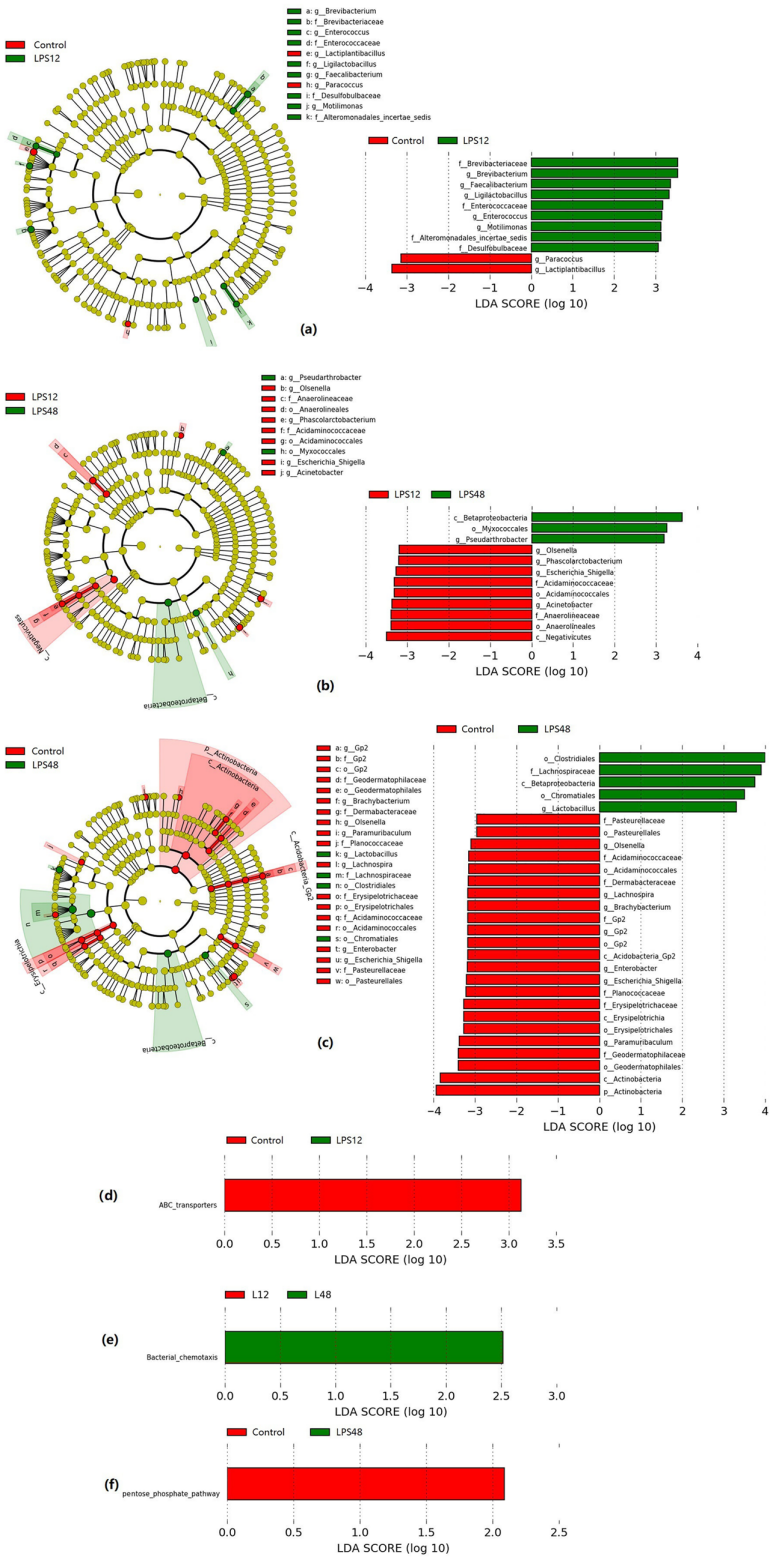
Linear discriminant effect size (LEfSe) analyses comparing differentially abundant taxa and Kyoto Encyclopedia of Genes and Genomes (KEGG) pathways between different groups. (**a**–**c**) Differences in the lung microbiota taxa between different groups. The phylogenic relationships between taxa with differences are shown in the cladogram. (**d**–**f**) Differences in the KEGG pathways predicted from the lung microbiota components between the different model groups.

Comparison of the Control and LPS12 groups by linear discriminant effect size (LEfSe) showed the presence of 11 taxa (including 7 genera) with significant differences (Fig. 5a). At the genus level, *Brevibacterium*, *Motilimonas*, *Enterococcus*, *Faecalibacterium*, *and Ligilactobacillus* were significantly increased in the LPS12 group versus the Control group, whereas *Lactiplantibacillus* and *Paracoccus* were significantly decreased.

In our comparison of the LPS12 and LPS48 groups, the abundances of a total of 12 taxa (related to 5 genera) were found to be significantly changed (Fig. 5b). At the genus level, *Pseudarthrobacter* was significantly increased in the LPS48 group as compared with the LPS12 group, whereas *Olsenella*, *Escherichia Shigella*, *Acinetobacter*, and *Phascolarctobacterium* were significantly decreased.

Finally, in our comparison of the Control and LPS48 groups, the abundances of a total of 28 taxa (including 8 genera) were found to be significantly changed (Fig. 5c). At the genus level, *Lactobacillus* was significantly increased in the LPS48 group as compared with the Control group, whereas *Paramuribaculum*, *Escherichia Shigella*, *Enterobacter*, *Gp2*, *Brachybacterium, Lachnospira*, and *Olsenella* were significantly decreased.

### Comparison of lung microbiota functionalities

We predicted a total of 6616 Kyoto Encyclopedia of Genes and Genomes (KEGG) orthologs (KOs) and 284 pathways from the datasets. LEfSe analysis in our comparison of between-group functionalities showed that the adenosine triphosphate–binding cassette (ABC) transporters pathway (ko02010) had significantly lower inferred abundances in the LPS12 group than in the Control group (Fig. 5d), the pentose phosphate pathway (ko00030) had significantly lower inferred abundances in the LPS48 group than in the Control group (Fig. 5e), and the bacterial chemotaxis pathway (ko02030) had significantly higher inferred abundances in the LPS48 group than in the LPS12 group (Fig. 5f).

### Analysis of correlation between lung microbiota and ALI/ARDS symptom indicators

**Figure 6:**
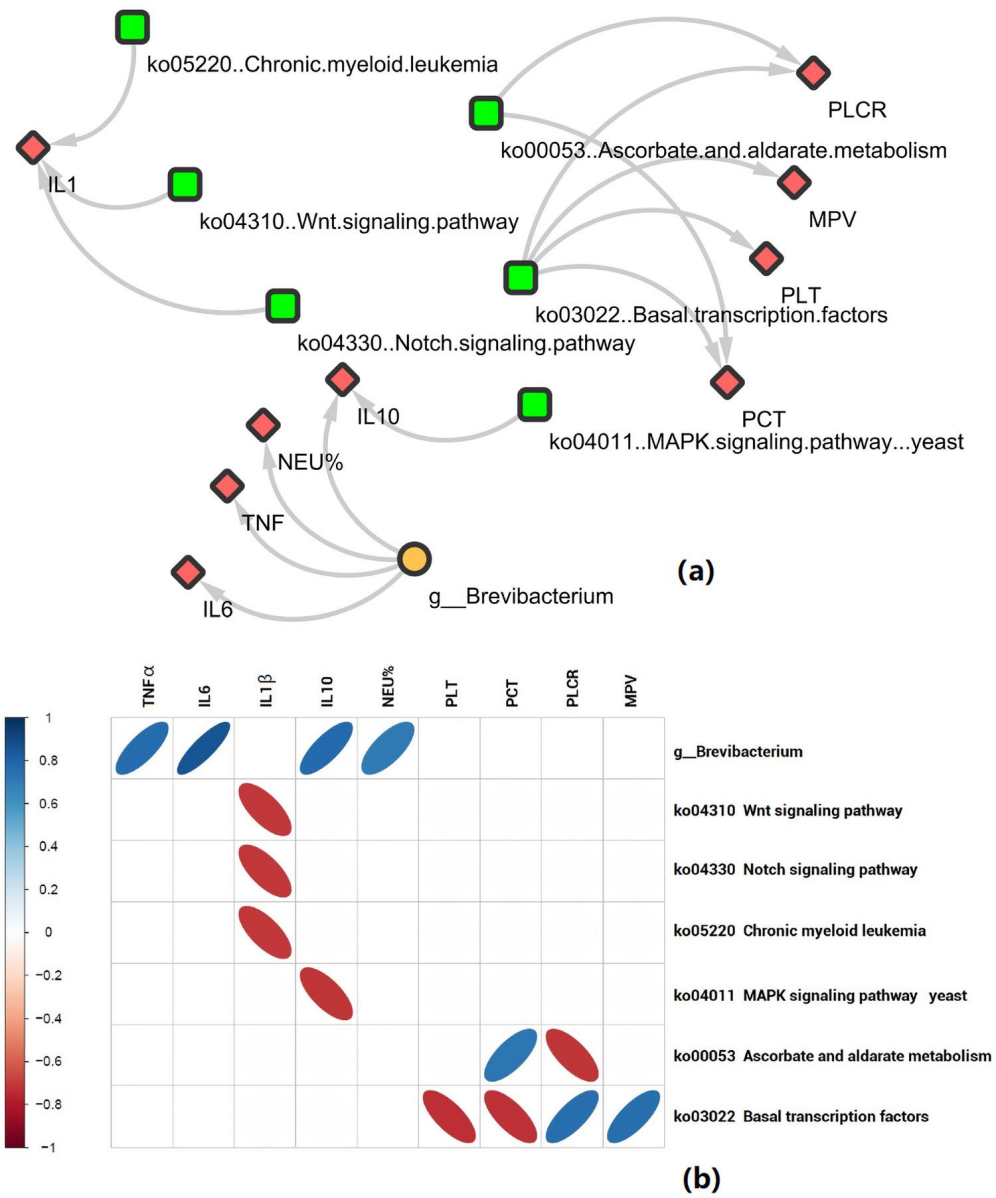
Correlations between host and microbiota parameters. (**a**) Host–microbiota interaction network. Network shows correlations between genus-level components and functionality pathways in lung microbiota and LPS-induced host ALI/ARDS symptoms from all samples. Lines between nodes represent correlations. Lines were drawn only for statistically significant correlations with absolute values of correlation coefficient > 0.70; unconnected nodes were omitted. (**b**) Spearman’s pairwise correlation. Blue = positive correlation; red = negative correlation. *NEU%* percentage of neutrophils, *MPV* mean platelet volume, *PLCR* platelet/large-cell ratio, *PLT* platelet count, *PCT* platelet packed volume.

To further assess potential correlations between LPS-induced ALI/ARDS symptoms and certain lung microbiota components or functionalities, we conducted Spearman’s correlation tests on genus-level and KEGG pathway relative abundance data using the psych package in R. The genus *Brevibacterium* and six functionality pathways were found to have significantly strong correlations (*P* < 0.05; correlation coefficient > 0.7 or < − 0.7) with LPS-induced ALI/ARDS symptoms (Fig. 6a). *Brevibacterium* was positively correlated with serum TNF-α, IL-10, and IL-6 levels and with hematological NEU%. In terms of microbiota functionalities, three signaling pathways—*Wnt* (ko04310), *Notch* (ko04330), and chronic myeloid leukemia (ko05220)—had negative correlations with IL-1β levels, while the mitogen-activated protein kinase (MAPK) signaling pathway–yeast (ko04011) was negatively correlated with IL-10 levels. Ascorbate and aldarate metabolism (ko00053) was positively correlated with PCT and negatively correlated with PLT. Finally, basal transcription factors (ko03022) were negatively correlated with PLT and PCT and positively correlated with PLCR and MPV (Fig. 6b).

## Discussion

Acute lung injury (ALI) is a common pulmonary response to a broad range of injuries or inflammation in the lungs or at other sites in the body. It is characterized by damage to the alveolar endothelial and epithelial barriers, recruitment of inflammatory cells, and onset of pulmonary edema^14^. ALI often leads to acute respiratory distress syndrome (ARDS), for which few effective therapeutic approaches exist to date^15^. Therefore, studies aimed at better understanding the pathogenic processes that develop in the injured lung are essential and might lead to the development of new methods to prevent and treat this disease.

The results of this study demonstrated that at different time points after LPS injection, along with fluctuations in systemic cytokine levels and the onset and regression of pulmonary edema, the diversity, components, and functionalities of the pulmonary microbiota in our rat model underwent significant dynamic changes. We also observed that several components and functionalities of these microbiota correlated with symptoms indicative of LPS-induced ALI.

Bacteria suspended in or attached to particles in the air are considered to be the main source of the lung microbiota. After the bacteria disperse into the lung, the microbiota is delicately balanced by bacterial reproduction rates and bacterial immigration and elimination^16^. Under normal circumstances, the rate of bacterial reproduction is low, while those of immigration and elimination are high^17^. We can therefore infer that under healthy conditions, the lung microbiota are highly diverse and affected by the surrounding environment. Indeed, this speculation is supported by a study by Dickson et al. on the lung microbiota of healthy mice, which demonstrated that this bacterial community is highly variable depending on cage, transportation, and supplier of mice and can better reflect the natural immune status of the host lung^18^. In lung disease, the balance of immigration and elimination is disturbed, resulting in bacteria that display competitive advantages to predominate, and thus, a decrease in diversity^19,20^.

In the present study, we observed that after LPS injection, the variation ranges of all three α-diversity indices were narrowed. These indices had all decreased significantly 48 h after LPS injection. These results, which were in line with those of the above-mentioned studies noting disturbance of the immigration/elimination balance and theorizing on the decrease in diversity, demonstrated the dynamic characteristics of changes to the microbiota diversity during systemic inflammation. In the early stage of inflammation (12 h after LPS injection), with the appearance of severe lung injury and edema, the host environment undergoes significant changes, such as increased levels of cytokine^21^ and endogenous antigenic peptides^22^. The influence of host factors on the lung microbiota was strengthened, overwhelming the influence of external environmental factors; this was reflected by the narrowing of the microbiota diversity index ranges within groups. In the later stage of inflammation (48 h after LPS injection), after the lung microenvironment had changed and competitive bacteria became dominant, α-diversity significantly decreased.

This decrease in lung bacterial diversity in the late stage of acute inflammation has been reported in other studies. For example, Poroyko et al*.* reported that the lung microbiota diversity was significantly reduced in a mouse model 72 h after LPS-induced ALI. They also demonstrated that the change in the lung microbiota could increase susceptibility to lung injury in model mice^5^. The association between lung microbiota diversity and lung diseases has also been reported in studies of other lung diseases, such as pneumonia, chronic obstructive pulmonary disease, cystic fibrosis (CF), and idiopathic pulmonary fibrosis, indicating that the diversity of the lung microbiota might play a critical role in bacteria–host interactions in the lung^6,23–26^.

However, until now, the compositional and functional basis of the lung microbiota diversity change remained to be clarified. In our comparisons of lung microbiota components and functionalities between different groups, we found that the component abundance varied drastically between groups, while changes in the functional abundance were relatively mild. A total of 51 taxa were found in our pairwise comparisons of species abundances in the three groups, but only three differential pathways were found in functionality abundance. The very high richness and diversity of the lung microbiota could be the cause of this phenomenon. A study analyzing the microbiota in different bodily sites of mice found a total of 226 genera in the lungs, but only 24 in the gut microbiota^27^. Similarly, using a more stringent method of selecting representative sequences (aligning reads to OTUs at a 100% rather than a 97% identity threshold) in the present study, we found 582 genera in the lungs as compared with only 38 genera in the rat gut microbiota in our previous study^28^. These findings all indicated that the lung microbiota contains many more species than other bodily habitats. In contrast to its components, the functionalities of the lung microbiota seem to be less variable and more stable. A total of 284 KEGG pathways were enriched from the lung microbiota in the present study—a number comparable to the 258 KEGG pathways enriched from the gut microbiota data in another study^29^.

In our functionality comparison between the Control and LPS12 groups, we found the ABC transporter pathway in lung bacteria to be significantly decreased in the LPS12 group. The ABC transporters are a family of transmembrane proteins that can transport a wide variety of substrates across biological membranes^30^. The ABC transporter protein is reported to be involved in the process of bacterial-iron acquisition and plays an important role in bacterial-iron competition^31^. Under physiological conditions, iron metabolism is tightly regulated to prevent iron toxicity. During infection, the host’s iron-scavenging activity level increases, and the response to inflammatory mediators leads to an increase in hepcidin, which can lead to hypoferremia^32^. Bacteria, like other cells, require iron for many basic cellular functions and metabolic pathways^33^, and therefore iron acquisition is essential for bacterial colonization in the competitive environment of the inflamed lungs. Studies have reported that in CF, lung bacteria adapt to and deploy specific systems based on the availability of iron, bioavailability of iron pools, stage of infection, and presence of competing bacteria^31^. Therefore, the change in the abundance of the ABC transporter pathway 12 h after LPS injection might reflect the adaptation of the lung bacteria to inflammatory conditions.

In our comparison of the LPS12 and LPS48 groups, we found that the bacterial-chemotaxis pathway in lung bacteria was significantly increased. This pathway is reportedly correlated with bacterial colonization on epithelial surfaces^34^. Tamar et al*.* showed that an acquired mutation in bacterial chemotaxis can change the inherent swimming behavior of bacteria that could barely reach the epithelial surface beyond the hydrogel mucosal layer, leading to a key enhancement in surface colonization^35^. Upregulation of the bacterial-chemotaxis function of the lung microbiota after 48 h of LPS infection might therefore have resulted from bacteria adapting to changes in an environment of edema.

Furthermore, in our comparison of the Control and LPS48 groups, we found the pentose phosphate pathway of the lung microbiota to be significantly decreased. The products of this pathway are known to be nicotinamide adenine dinucleotide phosphate (NADPH) and pentose phosphate. NADPH can provide hydrogen donors that are involved in a variety of metabolic reactions, preventing oxidation of various proteins and enzymes^36,37^, while pentose phosphate is necessary for nucleic acid biosynthesis. Studies have shown that nucleosides can increase the flux of the pentose phosphate pathway, thereby improving the effectiveness of DNA damage repair and improving cell survival^38^. Based on this evidence, we presumed that the reduction of the pentose phosphate pathway might reflect microbiota with lower antioxidant capacities and less efficient DNA damage repair ability after lung injury.

In this study, we also explored the correlation between physiological indicators that changed significantly during the development of ALI, as well as lung microbiota components and functionalities. We obtained a number of indicative results. In terms of microbiota composition, the abundance of the genus *Brevibacterium* was found to have a strong positive correlation with parameters, such as TNF, IL-6, IL-10, and NEU%. This genus is composed of organisms commonly found in the CF lung microbiome, as demonstrated in another study^39^. *Brevibacterium* has also been reported to be a causative agent of allergic alveolitis^40^. Our results were consistent with the above findings, suggesting that *Brevibacterium* might play a role in the progression of inflammation in the lungs.

In terms of lung microbiota functionality, the abundances of four signaling pathways—*Wnt* (ko04310), *Notch* (ko04330), chronic myeloid leukemia (ko05220), and MAPK–yeast (ko04011)—were found to have strong negative correlations with serum IL-1β and IL-10 levels, indicating that these pathways might play roles in the interaction between the lung microbiota and host immunity and should be addressed in more detail in follow-up studies. Finally, the abundances of the ascorbate and aldarate metabolism (ko00053) and basal-transcription factor (ko03022) pathways of the lung microbiota were found to be strongly correlated with four platelet-related physiological indicators: PLT, PCT, PLCR, and MPV. Current studies have found that platelets are important effectors of experimental ALI and clinical ARDS, playing diverse and complex roles in vascular-barrier integrity, organ repair, inflammation, activities across the immune continuum, and antimicrobial host defense^41^. The capacity of platelets to mediate protection or injury depending on conditions and context still needs further study^42^. The strong correlation we found between the two above-mentioned bacterial-function pathways and platelet-related indicators can provide clues for further elucidating the complex role of platelets in the occurrence and development of ALI.

In conclusion, this study will serve to provide greater insight into the dynamic changes in the diversity, components, and functionalities of the lung microbiota at different time points of lung injury. The representative differential components and functionalities of the lung microbiota at the time points we identified in this study will promote further understanding of the interaction between the host and the airway microbiota during lung injury and repair. Further exploration of the influence of the lung microbiota on host immunity and physiology might allow for better targeted approaches for the prevention and management of ALI and ARDS.

## Methods

### Animals and ethics statement

Eighteen male, 8-week-old, specific-pathogen-free (SPF) Sprague-Dawley rats weighing 200 ± 20 g were obtained from SPF Biotechnology Co., Ltd. (Beijing, China). Rats were housed in standard polypropylene shoebox cages (42 × 20.5 × 20 cm) on hardwood chip bedding in a designated room on alternate 12-h light/dark cycles at 24–26 °C and 50% humidity. They were allowed free access to water and fed a standard diet. All animal experiments were performed in accordance with Animal Research: Reporting of In Vivo Experiments (ARRIVE) guidelines. The study protocol was approved by the Ethics Committee of Minzu University of China (Beijing, China; No. ECMUC2020007AO). All experiments were performed in a Good Laboratory Practice (GLP)-accredited laboratory.

### Study design

Rats were randomly divided into three groups: Control (Control), LPS12, and LPS48. They were allowed to acclimatize to laboratory conditions for 7 days after arrival in the lab. The LPS12 and LPS48 groups received i.p. injections of LPS (*Escherichia coli 055*: 10 mg/kg body weight; Sigma-Aldrich, St. Louis, MO, USA), while the Control group received i.p. injections of a 0.9% NaCl solution. Rats in the LPS12 group were anesthetized 12 h after injection, and the Control and LPS48 groups were anesthetized with isoflurane 48 h after injection. Rats were sacrificed by exsanguination after blood was collected from the posterior vena cava. An aliquot of the whole blood from each mouse was centrifuged at 3000 rpm for 10 min, and the serum samples were stored at − 80 °C. The middle lobe of the right lung was dissected using sterile scissors and forceps immediately after opening the chest, and the lobes were placed in 5-ml sterile tubes containing 4 M guanidine thiocyanate solution as a preservative. The tubes were then stored at − 80 °C pending characterization of the lung microbiota. The upper lobe of the right lung was dissected, fixed in 10% formaldehyde solution, and subjected to histopathological examination. Finally, the left lung was dissected for lung W/D weight ratio calculation.

### Lung W/D weight ratio

Lung W/D weight ratio was measured to quantify pulmonary edema levels. The wet weight of the left lung was recorded immediately after dissection. The lung was then placed in an incubator at 80 °C for 48 h, and then the dry weight was recorded.

### Whole blood and serum analyses

A fully automatic blood cell analyzer (Sysmex, Tokyo, Japan) was used to detect hemoglobin (HB), red blood cells (RBCs), white blood cells (WBCs), platelets (PLTs), and other routine indicators. A total of 24 routine indicators were assessed. Serum inflammatory cytokines, including interleukin-1β (IL-1β), IL-6, IL-10, and TNF-α, were quantified using enzyme-linked immunosorbent assay (ELISA) kits (Shanghai Enzyme-linked Biotechnology Co., Ltd., Shanghai, China).

### Histopathological examination

Histological tissues were fixed in formalin for 12 h, cut into 5-μm thick sections, and stained with hematoxylin and eosin (H&E). The reagent used for histopathological examination was purchased from Wuhan Servicebio Technology Co., Ltd., Wuhan, China. The slides were de-identified and then observed by a pathologist.

An investigator blinded to group settings screened and analyzed 12–17 adjacent fields (× 100 objective magnification) covering the entire slide of each sample using ImagePro Plus software version 5.0 (Media Cybernetics, Silver Spring, MD, USA). The number of alveoli, sum of alveolar circumferences, and percentage of tissue area (red signal) per field were quantified for all screened images and further adjusted by the ratio of lung tissue area in each image. The tissue (red signal) and alveolar (white signal) areas were each set as hue (0–255) and saturation (0–255), and the intensities of the red and white signal areas were set at 165–255 and 0–164, respectively (Supplemental Fig. 1).

### Microbiota sequencing

Total bacterial deoxyribonucleic acid (DNA) was extracted using a Power Soil DNA Isolation Kit (MO BIO Laboratories, Inc., Carlsbad, CA, USA). After quality and quantity checks of the DNA sample at the ratios of 260/280 nm and 260/230 nm, the V3–V4 hypervariable region of the bacterial 16S ribosomal ribonucleic acid (rRNA) gene was amplified using the common primers 338F (5′-ACTCCTACGGGAGGCAGCAG-3′) and 806R (5′-GGACTACHVGGGTWTCTAAT-3′) combined with adapter and barcode sequences. Second-generation sequencing of purified, pooled polymerase chain reaction (PCR) sample products was performed on a HiSeq 2500 platform (Illumina, Inc., San Diego, CA, USA; 2 × 250 paired ends) at Biomarker Technologies Corporation (Beijing, China).

### Bioinformatic analyses

Raw sequence reads were processed using the UNOISE pipeline of the Usearch v11.0.667linux32 program (www.drive5.com/usearch/). High-quality sequences were classified into zero-radius operational taxonomic units (ZOTUs). The Ribosomal Database Project (RDP) classifier was then used to annotate taxonomic information of each ZOTU sequences with an 80% confidence threshold. The ZOTU table was sub-sampled randomly (to 43,683 reads, the smallest number of reads in the sample) to obtain equal sequencing depths among samples. The PD whole tree, Chao1, and Shannon indices were calculated using the QIIME 1.91 pipeline. Principal coordinates analysis (PCoA), distance-based redundancy analysis (db-RDA), and adonis tests were performed to evaluate differences among of bacterial communities based on Bray–Curtis distance metrics using “vegan” package in R. The KEGG-based orthologs and pathways functional profiles of the microbial communities were further predicted using the “Tax4Fun” R package^43^.

### Statistical analyses

The R software (version 3.52; R Foundation for Statistical Computing, Vienna, Austria) was used to perform all statistical analyses. A Shapiro–Wilk test was used to test for a normal distribution, and an analysis of variance (ANOVA) or Kruskal–Wallis test was used to evaluate differences in measured variables among the groups. Spearman’s *ρ* correlation analysis was performed on the associations between the different variables. Finally, LEfSe analysis (threshold of ± 2) was conducted to explore significant differences among the treatment groups using the relative abundance data of taxa and KEGG-based orthologs and pathways. P-values less than 0.05 were considered statistically significant.

### Ethics approval and consent to participate

This study was conducted with approval from the Ethics Committee of Minzu University of China (ECMUC2020007AO).

## Supplementary Information

Supplementary Figure 1.

## Data Availability

The datasets generated and/or analyzed during the current study are available in the SRA database repository, Accession No: PRJNA755451.
