# Supplementary figures and images for: Dynamic alterations in the lung microbiota in a rat model of lipopolysaccharide-induced acute lung injury
Source: Sci Rep. 2022 Mar 21;12:4791. doi: 10.1038/s41598-022-08831-8 (PMC8938502; doi:10.1038/s41598-022-08831-8)

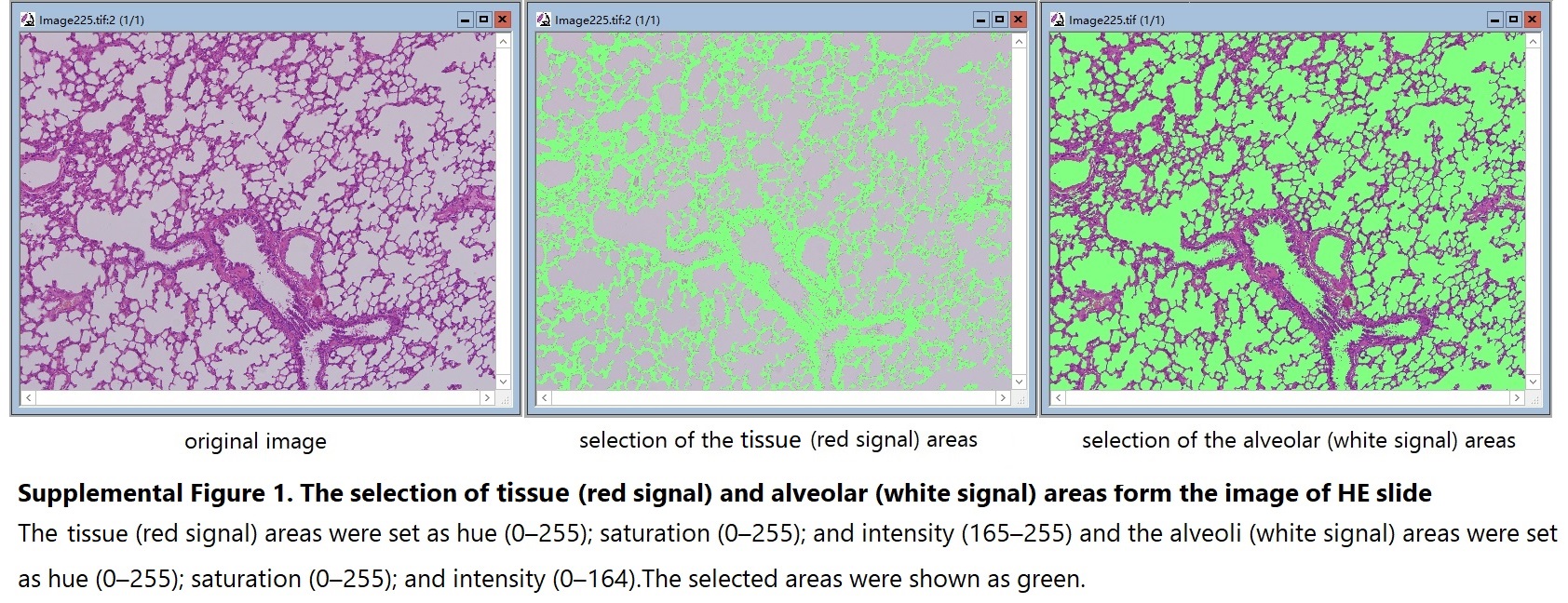

Supplement: Supplementary file 1 — Supplementary Figure 1. [file 41598_2022_8831_MOESM1_ESM.jpg]
